# An Update on the Quality of Life Measurements in Lung Cancer Patients Receiving Palliative Radiotherapy: A Literature Review

**DOI:** 10.4021/wjon591w

**Published:** 2013-05-06

**Authors:** Dominic Chu, Jasmine Nguyen, Kaitlin Koo, Liang Zeng, Gillian Bedard, Henry Lam, Erin Wong, Marko Popovic, Edward Chow

**Affiliations:** a Department of Radiation Oncology, Odette Cancer Centre, Sunnybrook Health Sciences Centre, University of Toronto, Toronto, Ontario, Canada

**Keywords:** Lung cancer, EORTC QLQ-C-30, Quality of life, Symptom palliation, Palliative radiotherapy

## Abstract

To conduct a systematic review on validated instruments used to assess quality of life (QOL) in patients with either primary or metastatic lung neoplasms. A literature search was conducted through the Embase (1950 - 2012 week 30) and Medline (1946 - 2012 week 3 July) databases. All compiled studies utilized QOL or symptom palliation as a primary or secondary outcome for patients with advanced lung cancer. A total of 17 studies met our criteria. Four questionnaires were most commonly used: the EORTC QLQ-C-30, the EORTC QLQ-LC-13, the Rotterdam Symptom Check-list (RSCL), and the Hospital Anxiety and Depression Scale (HADS). The limited number of studies assessing QOL in patients with advanced lung cancer suggests that QOL is still an uncommon endpoint for this patient population. Nine of seventeen (53%) studies evaluated QOL in their cohorts and out of those nine, seven (77%) included the use of a lung-specific tool. In total there were eleven of seventeen (65%) studies that evaluated symptom palliation, indicating the relevance of symptom palliation as an endpoint in this population. It is encouraged that lung specific QOL questionnaires, such as the FACT-L and the EORTC QLQ LC-13, be used in tandem with general questionnaires, such as the FACT-G and the EORTC QLQ C-30, in advanced lung cancer patients undergoing radiotherapy. Clinicians should also be advised to focus more on QOL assessment.

## Introduction

Lung cancer is the foremost cancer related cause of death in both men and women [1]. When diagnosed, 70% of lung cancers have already metastasized; common locations include the bones, liver, lymph nodes, and brain [1, 2]. The early stages of lung cancer are asymptomatic, which significantly contributes to its high morbidity and mortality rate [3]. Conversely, symptoms such as coughing, chest pain, dyspnea, weight loss, fatigue and chest infections become prevalent amongst patients in more advanced stages of lung cancer [3, 4]. These debilitating symptoms often warrant treatment in the form of radiotherapy, chemotherapy or a combination of the two [5]. Palliative radiotherapy plays a major role in the treatment of advanced lung cancer patients as it reduces endobronchial or extrinsic lesion size [6]. This decrease in lesion size is important in the alleviation of symptoms such as atelectasis, pain, cough, and shortness of breath, which in turn will gradually improve quality of life (QOL) [6, 7].

Treatments in the advanced lung cancer setting are often purely palliative in intent due to the progression of the disease. Hence, palliative endpoints such as QOL, that measure the overall well-being of a patient, may arguably be more important than traditional endpoints which have commonly focused on measures such as survival rates and disease-free survival. In addition, QOL has been shown to be a strong prognosticator of survival and tumour response in palliative settings, further emphasizing the need for prioritizing its use in clinical trials [8].

QOL assessment instruments have been developed by various organizations and have been integrated into patient care to assess physical, emotional, functional, and psychological well-being. Such questionnaires designed for lung cancer patients include the European Organization of Research and Treatment of Cancer (EORTC) Quality of Life Questionnaire - Lung Cancer (QLQ-LC 13), which is coupled with the general EORTC QLQ C-30 and the Functional Assessment of Cancer Therapy-Lung (FACT-L) which may be used in conjunction with the general FACT-G tool. Although the aforementioned tools remain the most commonly used, there are many variables to consider when choosing the best combination of such tools. This review aims to study these various validated QOL tools to help investigators decipher which tools are more appropriate in assessing advanced lung cancer patients undergoing palliative radiotherapy.

## Methods

### Search strategy

This literature review was performed on the OvidSP platform in Embase (1950 - 2012 week 30) and Medline (R) (1946 - 2012 week 3 July). Search terms: “lung cancer”, “advanced cancer”, “advanced (cancer or disease or adenocarcinoma or carcinoma or small cell or non-small cell)”, “metastasis”, “palliative radiotherapy” were combined with “quality of life”. Our more specific Medline search used the same keywords mentioned above, except with the term “lung neoplasm” rather than “lung cancer”.

### Inclusion and exclusion criteria

Studies that were included examined the assessment of QOL in patients diagnosed with lung cancer were treated with either palliative radiotherapy or brachytherapy and consisted of only retrospective/prospective cohort analyses or randomized trials. Included trials utilized QOL or symptom palliation as primary or secondary end-points. Case reports, literature reviews, and Non-English studies were excluded from this review. References within eligible articles were selected and examined by three co-authors.

### Data extraction

The following points were extracted from the included studies: 1) primary and secondary endpoints; 2) treatment details; 3) QOL or symptom assessment instruments (including performance status measure); 4) number of patients in each study arm and 5) median survival. Descriptive statistics summarized the findings.

## Results

A total of 775 articles were identified with 17 articles meeting the inclusion criteria. These seventeen articles evaluated palliative radiotherapy to the thoracic area in at least one study arm and assessed QOL or symptom palliation as either a primary or secondary endpoint.

Studies were categorized based on their patient samples and employed treatments. Ten studies examined patients with inoperable non-small-cell lung cancers (NSCLC) which were treated by external beam radiotherapy (supplementary data 1) (www.wjon.org). Four studies explored the use of endobronchial brachytherapy (EBB) in which radiation sources were placed at the site of the tumour, to treat symptomatic lung cancer (Table 1). Another four studies analyzed patients who had small-cell lung cancer (Table 2). One study by Stranzl et al had a cohort analysis which overlapped the characteristics of Table 1 and Table 2; it incorporated the use of EBB aimed to treat patients without NSCLC [9]. In some studies involving NSCLC patients, curative intent was indicated [7, 8], however the majority of studies examined the palliative population, for which QOL assessment was an integral tool for this population [10-17].

**Table 1:** Patients With Symptomatic Lung Cancer Treated With Endobronchial Brachytherapy (EBB) Compared to External Beam Radiotherapy With or Without EBB

*Stranzl et al, 2002 [9]	Prospective	Evaluate efficacy of Ir-192 HDR endobronchial brachytherapy for palliation of symptoms	15-20Gy/3-4fr in 5-6Gy once a week	11	Not stated	None	None	Study Designed: Subjective assessment of symptoms
Mallick et al, 2007 [11]	RCT	Testing endobronchial brachytherapy to improve QOL	A: 30Gy/10fr B: 10Gy/fr C: 15Gy/fr	114	Not stated	EORTC QLQ-C30 and QLQ-LC13 (2)	KPS	Study designed: physician graded symptom improvements
**Stout et al, 2000 [19]	RCT	Evaluate QOL outcomes of patients receiving palliative treatment	A: 15Gy/fr B: 30Gy/8fr	99	287 days (A), 250 days (B)	None	WHO	RSCL, HADS
Skowronek et al, 2011 [23]	Prospective	Demonstrate use and impact of brachytherapy	A: 22.5Gy/3fr B: 10Gy/1fr C: 7.5Gy/4fr	648	Not stated	None	KPS	None

*This study performed endobronchial brachytherapy on patients without non-small-cell lung cancer; ** This study uses both EBB as well as XRT. RCT: randomized controlled trial; TRT: thoracic radiotherapy; Gy: gray; fr: fractions; EORTC: European Organization for Research and Treatment of Cancer; NSCLC: non-small cell lung cancer; KPS: Karnofsky performance status; TOI: trial outcome index; MRC: Medical Research Council; FACT: Functional Assessment of Cancer Therapy; ECOG: Eastern Cooperative Oncology Group; RSCL: Rotterdam Symptom checklist; HDR: high dose rate.

**Table 2:** Patients With Inoperable Lung Cancer (Other Than NSCLC) Treated With Palliative Radiotherapy

Caissie et al, 2011 [10]	Retrospective	Assess QOL with EORTC C-15 PAL in radiotherapy patients	Not stated	29	Not stated	EORTC QLQ-C15-PAL (1)	KPS	None
Zeng et al, 2011 [18]	RCT	Report impact of fatigue in cancer patients	Not stated	500	Not stated	None	KPS	ESAS, Study designed: 4 grade scale to rate fatigue
Janda et al, 2004 [14]	RCT	Measuring QOL in radiotherapy patients	Not stated	288	Not stated	EORTC QLQ-C30	None	None

QOL: quality of life; KPS: Karnofsky performance status; fr: fractions; WHO: World Health Organization; EORTC: European Organization for Research and Treatment of Cancer; ECOG: Eastern Cooperative Oncology Group; HADS: Hospital Anxiety and Depression Scale; RSCL: Rotterdam symptom checklist; MRC: Medical Research Council; LCSS: Lung Cancer Symptom Scale; ESAS: Edmonton Symptom Assessment System; TOI: trial outcome index; FACT: Functional Assessment of Cancer Therapy; EBB: Endobronchial brachytherapy; XRT: external beam radiotherapy.

Studies were also grouped according to their primary endpoints. Twelve studies employed symptom palliation as the primary outcome [7, 10, 12, 13, 15-22], while thirteen of the identified trials used QOL [8, 10-17, 19-22]. Ten of the identified articles used both symptom palliation and QOL as primary outcomes [10, 12, 13, 15-17, 19-22]. Two of the studies used neither symptom palliation nor QOL as primary endpoints, but rather as secondary endpoints [9, 23].

### QOL and symptom palliation tools

A total of 9 validated tools were used in the literature to assess QOL or palliation of lung cancer related symptoms. The most commonly used validated tool was the EORTC QLQ-C30. In the QLQ C-30, five functional scales: physical, cognitive, emotional, and social functioning are included along with three symptom scales (fatigue, pain, and nausea and vomiting), as well as a specific item assessing financial impact [18]. In addition, this questionnaire considers symptoms that are prominent in patients with cancer including diarrhoea and dyspnea [3]. Each item on the test is scored on a numeric scale from 1 to 4 (1 = “not at all”; 4 = “very much”), while the last two items assessed overall health and overall QOL on a 1-7 scale (1= “very poor”; 7= “excellent”) [12]. Thus, for symptom scale items, higher scores represent a higher symptom burden, whereas on functional scale items (namely the last two items), higher scores represent higher functioning. Afterwards, scores from all single-item measures and scales may be linearly converted to a 0-100 scale. The EORTC QLQ-C30 was used in five of the seventeen studies [11, 12, 14-17]. The EORTC LC-13 was found in four articles and was used in conjunction with the EORTC QLQ-C30 three of those times as a lung cancer specific supplement [11, 12, 17]. The EORTC QLQ-C15 PAL was used in one trial without any lung cancer specific questionnaire [10]. This is a condensed version of the EORTC QLQ-C30 and is designed to relieve patient burden due to a large number of questionnaire items.

The FACT- L was used in two of the reviewed studies [13, 16]. It consists of general QOL questions that are divided into four categories: physical well-being, social/family well-being, functional well-being, and emotional well-being. The symptoms assessed include coughing, dyspnea, chest pain, weight loss, anorexia, and clarity of thought. These various subscales allow QOL to be divided into different components for analysis [13].

The Lung Cancer Symptom Scale was used in one study as a tool to measure six lung cancer specific symptoms and their effects on QOL: appetite, fatigue, cough, dyspnea, haemoptysis, and pain [20]. It uses a validated subjective rating scale that reports each item on a visual analog scale from 100 (best score) to 0 (worst score) [15].

The Edmonton Symptom Assessment System (ESAS) was used in one article and consists of six physical items (pain, fatigue, nausea, drowsiness, anorexia, and dyspnea) and three psychological symptom items (depression, sense of well-being, and anxiety) [18]. These items are measured on a ten point scale, zero indicating least distress and ten indicating the most distress [18]. There is evidence suggesting that the ESAS may be a good tool for screening the advanced cancer population as fatigue scores were similar with lengthy fatigue-specific tools [18].

The Rotterdam Symptom Check-list (RSCL) [7, 19, 21, 22] was employed in four studies, three of the four times in conjunction with the Hospital Anxiety and Depression Scale (HADS) [19, 21, 22]. The RSCL contained questions on a scale of 0-4 (0 = “no symptoms” and 4 = “severe symptoms”) that gave a subjective assessment of the severity of symptoms [19]. The HADS uses subscale scores of 0-7 to indicate normal mental health, 8-10 for borderline anxiety or depression and scores above 11 to indicate possible clinical anxiety or depression [15].

Lastly, the trial outcome index (TOI) was used twice as a measure of performance status as well as a prognostic tool for survival [13, 16]. It is also considered to be a strong measure of QOL as it is an alternative scoring of the FACT-L, which uses the sum of the FACT-L’s individual subscales [13]. It is a 21-item survey which takes into account the physical well-being, functional well-being, and lung cancer symptoms [16].

### Performance assessment

Fifteen out of the seventeen studies used a performance status (PS) assessment tool [7, 8, 10-19, 21-23]. PS was also included as a prognostic tool for patient survival. Four of the seventeen studies used a PS tool as a prognostic factor; three of the studies used performance tools for assessment of the patients. Karnofsky Performance Status (KPS) was the most utilized tool (six of seventeen studies), followed by the Eastern Cooperative Oncology Group (ECOG)/World Health Organization (WHO) which was found in six studies. Three of the studies did not use any tool to measure performance. There was one case of a clinician-assessed performance, as well as the use of the trial outcome index (TOI) as a supplement to measure PS as well as QOL.

## Discussion

In lung cancer palliative settings where traditional endpoints such as a cure and lengthened survival are unattainable, it is of the utmost importance to alleviate the patients’ burdens [8]. Effective palliative care must therefore focus on symptom control and maintenance of QOL [10]. Lung cancer presents many physical symptoms which include lack of energy, shortness of breath, coughing as well as psychological symptoms which include worrying and anxiety [23]. There are numerous treatment options for patients with advanced lung cancer, including chemotherapy, surgery, external beam radiotherapy, and endobronchial brachytherapy - however, palliative radiotherapy has been the most common treatment in reducing the severity of the various symptoms [5, 11].

When treated with palliative radiotherapy, patients will not only have to face progressive symptoms from the lung cancer itself, but the side-effects of the treatment as well [13]. It is therefore important to use a QOL measure when evaluating the effectiveness of treatment. In this case, QOL tools will allow insight on post-treatment options and provide more accurate information for clinical trials and for clinicians [13]. With regards to the treatment for this palliative population, the goal may be the stabilization rather than the improvement of QOL [13].

From the inconsistency in the use of QOL tools in our sample, it is clear that a consensus has still not been reached regarding which tools hold the highest priority in measuring the well-being of lung cancer patients. However, compared with the original review undertaken by Salvo et al [3], this update yielded a higher percentage in the use of QOL tools in conjunction with other tools, suggesting that the use of a combination of tools has shown increased precedence and perceived importance in clinical trials.

By comparing studies completed this decade - with the studies done before 2002 amidst the articles chosen in this present review, it is evident that QOL questionnaires have gained popularity. Among the 17 articles included in the present review, 75% of the studies done after 2002 used QOL assessment tools, while only 33% used QOL tools before 2002. By contrast, the studies chosen by Salvo et al show that only 37% of trials used QOL instruments after 2000 while 7% used QOL tools before 2000. These findings can be explained by the recent development of general QOL questionnaires. As these tools are reviewed and validated, clinicians may have become more inclined to use them and include them as an endpoint in clinical trials.

Equally noted was the parallel increase in the use of validated lung-specific tools. There were a total of six general QOL questionnaires in this study; four of those (67%) used a validated lung-specific QOL tool. In contrast, Salvo et al, found twelve general QOL questionnaires, where six (50%) of those used lung-specific QOL questionnaires. A positive trend in the use of such tools has thus become evident suggesting that more studies have prioritized the use of validated lung-specific questionnaires in conjunction with general questionnaires to evaluate QOL in patients with advanced lung cancer. This tandem use of questionnaires allows for easier comparison among trials, may increase the validity of the each study, and allows for greater consistency in evaluation of QOL through examination of lung specific symptoms such as haemoptysis, dyspnea, and cough [3, 8]. Although the use of QOL tools has increased, this review found a smaller percentage of the use of QOL tools on their own compared what was found by Salvo et al [3]. Within our review, 18% of trials measured QOL using only one tool, while Salvo et al had 30% of its studies measuring QOL with the use of only one tool. The negative trend was also apparent within the trials measuring solely symptom palliation. In contrast to this trend, the percentage of the use of both QOL and symptom palliation assessments increased from 14% (as found by Salvo et al) to nearly 53% in this update. This increase is supported by the findings of Mallick et al who discovered a strong correlation with the improvement of QOL and symptom palliation assessment after a month of endobronchial radiotherapy [11]. This indicates and suggests the importance of the use of both QOL and symptom palliation in conjunction with one another.

Stout et al [19] compared the use of patient and clinician assessments of symptom palliation; a major disparity was found as doctors had severely underestimated symptoms such as breathlessness, anorexia, tiredness, and nausea. The initial emphasis on symptom palliation gave clinicians inaccurate information towards their study, which then led to the suggestion of using QOL analysis, rather than symptom palliation in the evaluation of treatments [19]. This does not rule out the use of symptom palliation assessment, but advocates for the inclusion of QOL assessment in addition to assessment of symptom palliation. The promotion of QOL assessment is consistent with and is supported by Auchter et al who mentions that QOL questionnaires such as the FACT-L have a lack of correlation between toxicity grade (measured by symptoms) and QOL [16]. This is reflected in an improvement in QOL usage among studies by from Salvo et al. (44%) compared to this updated literature review (53%).

QOL assessment however does have compliance issues. This was noticed over time as patients’ health deteriorated [12, 13]. Initially the compliance rate was usually in the high 90th percentile, even with advanced malignancies [12]. Heyes shows that this rate drops to 50% over a span of 4 months [13]. This trend of a high compliance from the first assessment to a low compliance months later was noted in other studies [12, 13, 17]. Despite its significant declining compliance rate, the EORTC QLQ-C30 performed consistently well with the various studies [11-13, 16, 17, 22]. Caissie et al have demonstrated that the EORTC QLQ-C15-PAL may be equally effective in the measurement of QOL, but would decrease the burden on due to questionnaire length and thereby improve patient compliance [8]. Only one study in this review used the EORTC QLQ-C15-PAL and none used it in the studies found by Salvo et al, as it is still not fully recognized as a universal core questionnaire to assess QOL. It is encouraged for investigators to use the EORTC QLQ-C15-PAL in future trials [8].

Within this review, a few limitations exist. Subjective judgement was needed on several of the evaluated criteria including QOL, symptom severity, and symptom improvement. Some of this was through the use of study designed questionnaires created by individual investigators. In addition, only English articles were selected for this review. Another limitation in this study is that only 17 articles were found, of which 9 measured QOL in cohorts.

In conclusion, the continued and increased use of QOL assessment is critical in the treatment of advanced lung cancer patients undergoing palliative radiotherapy. It is recommended that future clinical trials involve the use of a general cancer questionnaire coupled with a specific lung cancer questionnaire. In addition, future trials should prioritize the assessment of QOL over the measurement of symptom palliation - it is important to note that the exclusion of the measurement of symptom palliation is not suggested.

**Table 3:** Frequency of Instruments Used in Clinical Trials Measuring Quality of Life (QOL) in Patients With Locally Advanced Lung Cancer or Lung Metastases

Instrument	Frequency
European Organization for Research and Treatment of Cancer (EORTC)	
General cancer questionnaire (EORTC QLQ - C30)	5
Lung cancer questionnaire (EORTC QLQ - LC-13)	4
General cancer questionnaire (EORTC QLQ - C15 PAL)	1
Functional Assessment of Cancer Therapy (FACT)	
Lung questionnaire (FACT - L)	2
Rotterdam Symptom Check-list	4
Hospital Anxiety and Depression Scale (HADS)	3
Trial Outcome Index (TOI)	2
Lung Cancer Symptom Scale (LCSS)	1
Edmonton Symptom Assessment System (ESAS)	1
